# Collective Activity Bursting in a Population of Excitable Units Adaptively Coupled to a Pool of Resources

**DOI:** 10.3389/fnetp.2022.841829

**Published:** 2022-03-28

**Authors:** Igor Franović, Sebastian Eydam, Serhiy Yanchuk, Rico Berner

**Affiliations:** ^1^ Scientific Computing Laboratory, Center for the Study of Complex Systems, Institute of Physics Belgrade, University of Belgrade, Belgrade, Serbia; ^2^ Neural Circuits and Computations Unit, RIKEN Center for Brain Science, Wako, Japan; ^3^ Institut für Mathematik, Technische Universität Berlin, Berlin, Germany; ^4^ Potsdam Institute for Climate Impact Research, Potsdam, Germany; ^5^ Institut für Mathematik, Humboldt-Universität zu Berlin, Berlin, Germany; ^6^ Institut für Physik, Humboldt-Universität zu Berlin, Berlin, Germany; ^7^ Institut für Theoretische Physik, Technische Universität Berlin, Berlin, Germany

**Keywords:** local and collective excitability, heterogeneous neural populations, metabolic resources, collective bursting, adaptive coupling, switching dynamics, multiscale dynamics, multistability

## Abstract

We study the collective dynamics in a population of excitable units (neurons) adaptively interacting with a pool of resources. The resource pool is influenced by the average activity of the population, whereas the feedback from the resources to the population is comprised of components acting homogeneously or inhomogeneously on individual units of the population. Moreover, the resource pool dynamics is assumed to be slow and has an oscillatory degree of freedom. We show that the feedback loop between the population and the resources can give rise to collective activity bursting in the population. To explain the mechanisms behind this emergent phenomenon, we combine the Ott-Antonsen reduction for the collective dynamics of the population and singular perturbation theory to obtain a reduced system describing the interaction between the population mean field and the resources.

## 1 Introduction

Complex dynamical networks are indispensable for modeling many processes in nature, technology, and social sciences (Strogatz, 2001; Boccaletti et al., 2006; Arenas et al., 2008; Yanchuk et al., 2021). In realistic situations, collective dynamics in such networks is affected by the constraints on available resources from the environment (Roberts et al., 2014; Kroma-Wiley et al., 2021), resulting in complex dynamical phenomena, especially if the systems are self-organized to operate close to criticality (Levina et al., 2007). Often, additional resource dynamics gives rise to adaptive mechanisms such as frequency adaptation (Fuhrmann et al., 2002; Taylor et al., 2010; Ha and Cheong, 2017; Kroma-Wiley et al., 2021), delay adaptation (Fields, 2015; Park and Lefebvre, 2020), or various forms of homeostatic plasticity in neuronal systems (Zierenberg et al., 2018).

Compared with other somatic cells, neurons have a very high energy consumption (Attwell and Laughlin, 2001) and are highly sensitive to energy limitations affecting their cellular metabolic processes. Hence, the availability of metabolic resources, their dynamics and their interplay with the neuronal activity are important factors for the overall performance of neural networks and their homeostasis (Vergara et al., 2019). Dynamical networks with resource constraints have been in the focus of recent studies (Taylor et al., 2010; Roberts et al., 2014; Virkar et al., 2016; Nicosia et al., 2017; Song et al., 2020; Kroma-Wiley et al., 2021). In particular, in (Song et al., 2020) it has been investigated how phase synchronization between the mutually uncoupled system elements depends on the interaction with the environment. A mini-review (Roberts et al., 2014) has highlighted the importance of reciprocal coupling between neuronal activity and metabolic resources in self-organizing and maintaining neuronal operation near criticality, and has also presented a general slow-fast formulation for the case where resources change slowly relative to neural activity. In (Virkar et al., 2016), a discrete two-layer model has been proposed to describe a mechanism by which metabolic resources are distributed to neurons via glial cells. An example of frequency adaptation in Kuramoto model was provided in (Taylor et al., 2010), reproducing certain phenomena that are not qualitatively accounted for the classical Kuramoto model, such as long waiting times before reaching synchronization. In (Nicosia et al., 2017), neuronal dynamics and nutrient transport were assumed to be bidirectionally coupled, such that the allocation of the transport process at one layer depends on the degree of synchronization in the other and vice versa. In (Kroma-Wiley et al., 2021), a system of coupled Kuramoto oscillators that consume or produce resources depending on their oscillation frequency was considered.

Inspired by the mechanisms for the interaction of a neuronal network with a population of glial cells, the studies (Fields, 2015; Lücken et al., 2017; Park and Lefebvre, 2020) introduced models of networks with adaptive time-delays.

Of particular interest are adaptive networks in which connectivity changes are related to intrinsic nodal dynamics (Gross and Blasius, 2008; Berner, 2021). For example, these types of networks can model synaptic neuronal plasticity (Meisel and Gross, 2009; Markram et al., 2011), chemical (Jain and Krishna, 2001; Kuehn, 2019), epidemic (Gross et al., 2006), biological, and social systems (Horstmeyer and Kuehn, 2020). A paradigmatic example of adaptively coupled phase oscillators gained considerable interest recently (Gutiérrez et al., 2011; Kasatkin et al., 2017; Berner et al., 2019a; Berner et al., 2019b; Berner et al., 2020; Feketa et al., 2020; Berner et al., 2021). This type of phase oscillator models seems to be useful for predicting and describing phenomena in more realistic and detailed models (Popovych et al., 2015; Lücken et al., 2016; Röhr et al., 2019) as well as for the understanding of collective phenomena such as multicluster states (Berner et al., 2019a; Berner et al., 2019b) or recurrent synchronization (Thiele et al., 2022).

In the present paper, we consider coupled excitable units (Lindner et al., 2004; Izhikevich, 2007), characterized by a linearly stable rest state susceptible to finite-amplitude perturbations. Excitable systems act as nonlinear threshold-like elements, such that applying a sufficiently small perturbation gives rise to a small-amplitude linear response, while a perturbation exceeding a certain threshold may trigger a large-amplitude nonlinear response. A classical example for the excitability feature are neurons (Ermentrout and Kopell, 1986; Izhikevich, 2007) which respond to a supra-threshold stimulation by emitting a spike. Apart from neuronal systems, excitability is important for other living cells (Scialla et al., 2021), lasers (Yanchuk et al., 2019; Terrien et al., 2021), chemical reactions (Chigwada et al., 2006), machine learning (Ceni et al., 2019), and many other fields. A variety of phenomena, including resonances, oscillations, patterns and waves, are caused by the interplay of excitability and noise (Pikovsky and Kurths, 1997; Neiman et al., 1999; Pototsky and Janson, 2008; Franović et al., 2015; Bačić et al., 2018a; Bačić et al., 2018b; Franović et al., 2018; Zheng and Pikovsky, 2018; Bačić and Franović, 2020; Franović et al., 2020) or time-delay (Brandstetter et al., 2010; Klinshov et al., 2016).

As a prototype of excitable local dynamics, we consider active rotators, paradigmatic for type I excitability (Shinomoto and Kuramoto, 1986; Park and Kim, 1996; Lindner et al., 2004; Osipov et al., 2007; Dolmatova et al., 2017; Franović et al., 2020; Klinshov et al., 2021). Active rotators have been used to study interacting excitable systems with noise (Lindner et al., 2004), synchronization in the presence of noise (Shinomoto and Kuramoto, 1986; Park and Kim, 1996; Dolmatova et al., 2017; Klinshov et al., 2021), the interplay of noise and an adaptive feedback (Franović et al., 2020), effects of an adaptive network structure (Thamizharasan et al., 2021), co-effects of noise, coupling, and adaptive feedback (Bačić et al., 2018b; Song et al., 2020) or delayed feedback (Yanchuk et al., 2019) and the impact of higher-order Fourier modes (Ronge and Zaks, 2021), to name but a few.

An important ingredient of our model is the multiscale structure of the dynamics, whereby the processes at the pool of resources are assumed to occur much slower than the dynamics of excitable units at the nodes. Utilizing this feature, we apply the methods of singular perturbation theory (Desroches et al., 2012; Kuehn, 2015) to first study the fast dynamics (layer dynamics) for fixed resource levels with the Ott-Antonsen approach, and then reduce the problem to the slow dynamics of resources.

Our main result consists in demonstrating how the adaptive interaction between a population of excitable units with a pool of resources gives rise to collective activity bursting. Such emergent dynamics is characterized by alternating episodes of stationary and oscillating behavior of the macroscopic order parameter. We describe the mechanisms behind the activity bursting and indicate parameter regions where this phenomenon can be reliably observed. So far, collective bursting phenomena have been considered to emerge due to time-varying neuronal inputs (Stoop et al., 2002), the interplay of external input and homeostatic plasticity (Zierenberg et al., 2018), or synaptic short-term plasticity (Gast et al., 2020). In these studies, possible implications for healthy and diseased brain states have been drawn. Moreover, the important role of bursting phenomena for the understanding of brain-organ interactions have been highlighted in the perspectives article (Ivanov, 2021). Our study complements recent research on emergent bursting dynamics in brain and organ systems by providing a simple and analytically tractable model generating collective activity bursting.

Our paper is organized as follows. In Section 2 we lay out the model of a heterogeneous population of excitable units adaptively coupled to a pool of resources, while in Section 3 we introduce the main phenomenon of collective activity bursting. Section 4 and Section 5 concern the analysis of the system’s multiscale dynamics within the framework of singular perturbation theory, first elaborating on the layer problem and then using the reduced problem to explain the mechanism of collective bursting and the origin of multistability in the full system. Section 6 proposes two different approaches to induce switches between the coexisting collective regimes, whereas Section 7 provides our concluding remarks and outlook.

## 2 Model

We consider a system of *N* coupled active rotators (Strogatz, 1994) with a Kuramoto-type coupling given by,

$${\dot{\phi }}_{k}=I_{k}\left(r\right)-\sin\phi_{k}+\frac{\sigma }{N}\overset{N}{\underset{j=1}{\sum }}\sin\left(\phi_{j}-\phi_{k}\right),$$ (1)

where *ϕ*_*k*_ ∈ [0, 2*π*), *k* = 1, … , *N* are the local phase variables, and *σ* is the coupling strength. While providing a simplified description of local dynamics, active rotators manifest the excitability feature crucial to neuronal activity (Strogatz, 1994; Izhikevich, 2007), and are similar to the model of theta neurons (Luke et al., 2013; Laing, 2014) paradigmatic for type I neural excitability. Note that more detailed models of neuronal dynamics, such as those of Morris-Lecar (Morris and Lecar, 1981) and Wang-Buzsáki (Wang and Buzsáki, 1996), also belong to this excitability class. External inputs *I*_*k*_(***r*** (*t*)) = *r*_1_ (*t*) + *r*_2_ (*t*) *ν*_*k*_ received by each unit comprise of a *homogeneous* component *r*_1_ (*t*), acting identically at all the units, and a *heterogeneous* component, where the variability is due to parameters *ν*_*k*_ drawn from a normalized Gaussian distribution $\nu_{k}\in N\left(0,1\right)$ . Recall that in models of coupled active rotators, terms *I*_*k*_ are classically interpreted as local bifurcation parameters describing individual oscillation frequencies. Nevertheless, here *I*_*k*_ (*t*) at each moment follow a Gaussian distribution $g\left(I\right)=N\left(r_{1},r_{2}^{2}\right)$ , such that the local velocities of the units are modulated by coupling to *r*_1_ and *r*_2_. The latter modulation can be seen as describing an interaction with the *resources* from the environment (Song et al., 2020; Kroma-Wiley et al., 2021) summarized within the two-component resource variable ***r*** = (*r*_1_, *r*_2_). In the context of neuroscience such modulation of local velocities is reminiscent of frequency adaptation of neuronal spiking (Fuhrmann et al., 2002; Ha and Cheong, 2017) due to a limited amount of metabolic resources affecting e.g. neurotransmitters.

Adaptation of spiking activity is a slow process compared to spike emission (Ha and Cheong, 2017), which should be reflected in the dynamics of metabolic resources ***r*** (*t*). In fact, a model involving such a separation of time scales has recently been proposed to describe the interplay of energy consumption and activity in neuronal populations (Roberts et al., 2014). Here we introduce a simple model of dynamical resources based on the Hopf normal form. We consider ***r*** as a complex variable, i. e, ***r*** = *r*_1_ + i*r*_2_, which satisfies the dynamical equation

$$\dot{r}=\epsilon f\left(r-s,\lambda \right),$$ (2)

$$\dot{\lambda }=-\epsilon^{\prime }\left(\lambda -\lambda_{0}-\gamma A\left(t\right)\right),$$ (3)

with activity

$$A\left(t\right)≔\frac{1}{N}\overset{N}{\underset{j=1}{\sum }}{\dot{\phi }}_{j},$$ (4)

The metabolism describing function given by *f* (***r***, λ) = ***r*** (λ + i*ω* − | ***r*** |^2^), the frequency *ω* and the resource base level given by ***s*** = *s*_1_ + i*s*_2_. Small parameters *ϵ* ≪ 1 and *ϵ*′ ≪ 1 are introduced to account for the scale separation between the fast spiking dynamics of units and the slowly adapting dynamics of the resources. Note that we consider the case *ϵ*′ = *ϵ* throughout the paper.

System Eq. 2, 3 that describes the interaction between the two resources undergoes a supercritical Hopf bifurcation at λ = 0. This allows for the interpretation of the resource dynamics as being *inactive* if λ < 0, when it possesses a stable focus at ***s***, or as *active* if λ > 0, when it displays a stable limit cycle. In other words, in the inactive states, the resource dynamics lies stationary at the resource base level ***s*** = *s*_1_ + i*s*_2_, while for active states, the resource dynamics is attracted to a periodic orbit that encircles the resource base level. We further assume that the dynamics of metabolic resources adapts to the activity *A* (*t*) of the population, see Eq. 4. In particular, the adaptation dynamics Eq. 3 can be regarded as a feedback mechanism whereby due to a feedback loop, an activated neuronal population may activate the pool of resources which in turn may further activate or even deactivate the neuronal population. The adaptation strength is described by parameter *γ* which controls the impact of the population’s dynamics on the dynamics of resources. Throughout the paper, we keep *γ* = 0.5. In case of no spiking activity, i.e., if *A* (*t*) = 0 or *γ* = 0, the resource dynamics is inactive and the corresponding resource activity variable λ settles to the rest level λ_0_. In the remainder, the level λ_0_ = −0.05 is assumed to correspond to a stable steady state at ***s***. Due to the dynamical interplay between the metabolic resources and the neuronal population, the activity variable *λ* (*t*) may change in time. Accordingly, the state of the resources may change between active (periodic attractor) and inactive (stationary state). To describe the coherence of the population dynamics, we use the complex order parameter *Z* defined by

$$Z\left(\phi \left(t\right)\right)=\frac{1}{N}\overset{N}{\underset{j=1}{\sum }}e^{\text{i}\phi_{j}\left(t\right)}=R\left(\phi \left(t\right)\right)e^{\text{i}\Theta \left(\phi \left(t\right)\right)},$$ (5)

where *R* is the Kuramoto order parameter, and Θ is the mean phase (Bick et al., 2020).

**FIGURE 1:**
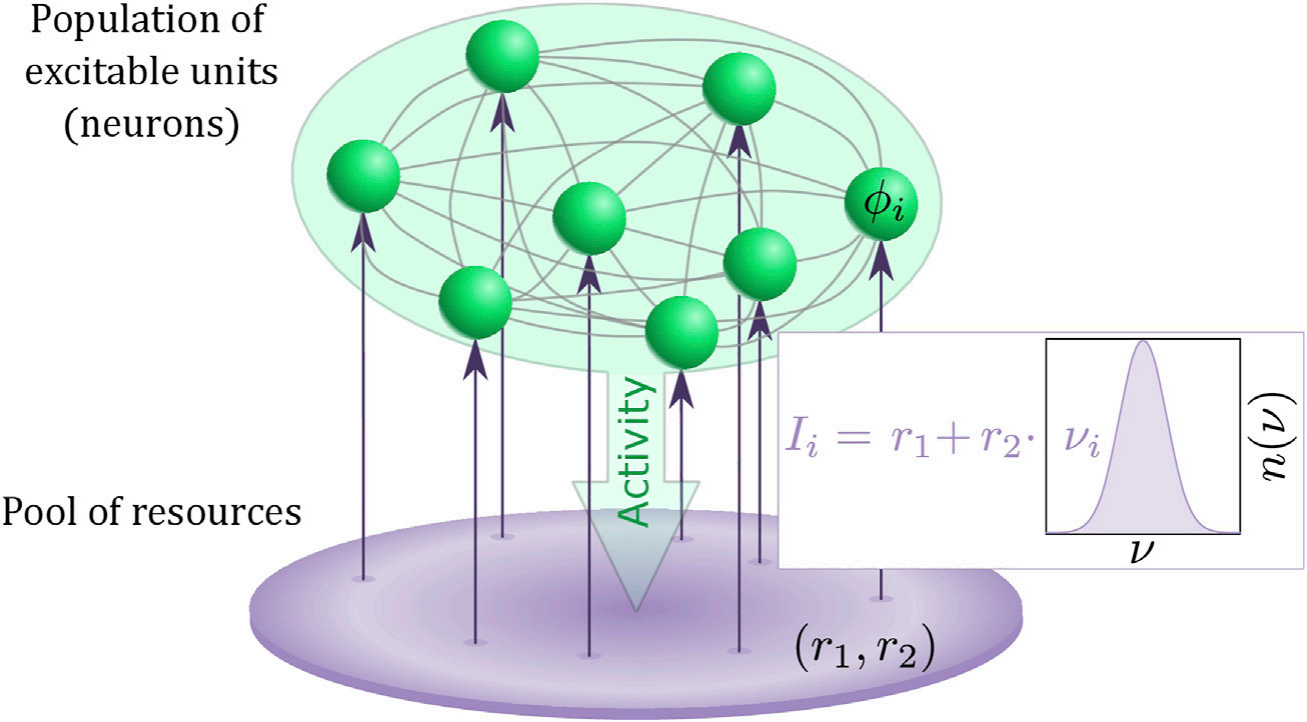
Schematic for the two-layer model consisting of a heterogeneous population of excitable units (green) and interacting pool of resources described by an adaptive Stuart-Landau oscillator (purple). The heterogeneity *ν*_*i*_ of the excitable units are randomly drawn from a distribution *n* (*ν*).

Summarizing, we have proposed a multiscale model of a heterogeneous population of active rotators, featuring local excitability and spike frequency adaptation as two important ingredients of typical neuronal activity, coupled to a pool of resources that slowly adjusts its dynamics to the activity of the population. Figure 1 provides an illustration of our model.

## 3 Collective Activity Bursting

In this section, we briefly introduce the phenomenon of collective activity bursting induced by an adaptive coupling to resources. A more detailed analysis of the phenomenon will be performed in the subsequent sections.

**FIGURE 2:**
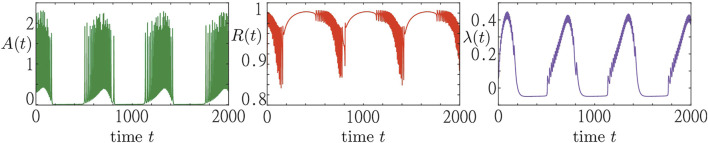
Collective activity bursting in system Eqs 1–3. Three panels show the time traces of the population activity *A* (*t*) (green), the order parameter *R* (*t*) (red) and the resource activity variable *λ* (*t*) (blue) from left to right, respectively. The trajectory is obtained from a random initial condition for a system of *N* = 5,000 active rotators and parameters: *σ* = 5, *ϵ* = 0.05, *s*_1_ = 0.97, *s*_2_ = 1.2, *ω* = 0.2, *λ*_0_ = −0.05, *γ* = 0.5.

In Figure 2, we show a simulation result of a system consisting of *N* = 5,000 active rotators adaptively coupled to a pool of resources as described by Eqs 1–3. The emergent collective dynamics within the population is represented by the macroscopic variables *A* (*t*) and *R* (*t*). The dynamics within the resource pool is characterized by the activity variable *λ* (*t*). We observe that the population of active rotators displays a recurrent temporal formation of bursts in the macroscopic activity *A* (*t*) followed by periods of inactivity. Such episodes of macroscopic activity and inactivity correspond to episodes of a rapidly and slowly varying order parameter *R* (*t*), respectively. Switching between the different regimes is equally well visible in the evolution of the resource variable *λ* (*t*) showing the pattern of recurrent activation (*λ* > 0) and deactivation (*λ* < 0).

We note that this recurrent switching between macroscopic activity and inactivity is due to the adaptive feedback provided by the dynamical resources and can not be observed in a system of active rotators alone. In fact, active rotators are a paradigmatic model for excitable systems, supporting regimes of either activity ${\dot{\phi }}_{i}>0$ or inactivity ${\dot{\phi }}_{i}=0$ depending on parameters such as the input currents *I*_*i*_, see e.g. (Franović et al., 2020) for more details. The slow adaptation of the input currents caused by the resource dynamics, however, provides a mechanism to switch between the two regimes. In the following sections, we systematically describe the emergence of collective activity bursting by making use of the separation of timescales between the dynamics of the population and the resources. The slow-fast analysis within singular perturbation theory, see e.g. (De Maesschalck and Wechselberger, 2015; Kuehn, 2015), allows for a splitting of multiscale dynamics into a so-called layer dynamics of the fast variables and an averaged dynamics for the slow variables.

The layer dynamics of system Eqs 1–3 consists of a population of actively coupled rotators with input currents drawn for a Gaussian distribution $N\left(r_{1},r_{2}^{2}\right)$ . The subsequent analysis of the layer equation in Section 4 provides us with a clear mapping for the regimes of population activity and inactivity. Building on this, we analyse the full system Eqs 1–3 and show that the collective activity bursting emerge close to criticality, i.e., the boundary between activity and inactivity of the layer dynamics. We also describe regimes of multistability between activity bursting and inactivity, and provide insights into perturbations that give rise to transitions between different states.

## 4 Layer Dynamics: Heterogeneous Population of Active Rotators

The fast subsystem, describing the evolution of the original slow-fast problem Eqs 1–3 on the fast timescale, comprises of a heterogeneous assembly of *N* globally coupled active rotators

$$\dot{\phi_{k}}=r_{1}+r_{2}\nu_{k}-\sin\phi_{k}+\frac{\sigma }{N}\overset{N}{\underset{j=1}{\sum }}\sin\left(\phi_{j}-\phi_{k}\right).$$ (6)

In the absence of adaptation of the resource variables *r*_1_ and *r*_2_, the local dynamics ${\dot{\phi }}_{k}=I_{k}-\sin\phi_{k}$ depends on external input *I*_*k*_ = *r*_1_ + *r*_2_ *ν*_*k*_ which may be seen as an effective bifurcation parameter mediating the transition between an excitable (*I*_*k*_ ≲ 1) and oscillatory regime (*I*_*k*_ > 1) via a SNIPER (saddle-node infinite period) bifurcation at |*I*_*k*_| = 1. In the singular limit *ϵ* → 0, system (Eq. 6) defines the layer problem, where *r*_1_ and *r*_2_ are treated as additional system parameters.

According to classical singular perturbation theory (De Maesschalck and Wechselberger, 2015; Kuehn, 2015), the layer problem describes solutions of the multiscale system Eqs 1–3 on a timescale much shorter than 1/*ϵ*, where the variables *r*_1_ and *r*_2_ do not change significantly. In particular, it can describe fast (rapidly changing) segments of the solutions.

### 4.1 Ott-Antonsen Approach for the Layer Dynamics

We analyze the layer problem by determining the stability of stationary solutions of the layer dynamics and their bifurcations within the framework of Ott-Antonsen theory (Ott and Antonsen, 2008; Ott and Antonsen, 2009). We start by rewriting the layer dynamics in terms of complex order parameter (Eq. 5), which leads to

$${\dot{\phi }}_{k}=I_{k}-\sin\phi_{k}+\sigma Im\left(Z\left(t\right)e^{-i\phi_{k}}\right).$$ (7)

In the thermodynamic limit *N* → *∞*, the state of the population can be described by the probability density *h* (*ϕ*, *I*, *t*), which satisfies the normalization condition $\int_{0}^{2\pi }h\left(\phi ,I,t\right)d\phi =g\left(I\right)$ , see e.g. (Omel’chenko and Wolfrum, 2012; Omel’chenko and Wolfrum, 2013). The continuity equation for *h* (*ϕ*, *I*, *t*) then reads

$$\frac{\partial h}{\partial t}+\frac{\partial }{\partial \phi }\left(hv\right)=0,$$ (8)

where the velocity is given by *v* = *I* − sin *ϕ* + *σ*Im (*Z*(*t*)*e* ^−*iϕ*^). According to Ott-Antonsen ansatz (Ott and Antonsen, 2008; Ott and Antonsen, 2009), the long-term dynamics of Eq. 8 settles onto an invariant manifold of the form

$$h\left(\phi ,I,t\right)=\frac{g\left(I\right)}{2\pi }\left\{1+\overset{\infty }{\underset{n=1}{\sum }}\left[{\bar{z}}^{n}\left(I,t\right)e^{\text{i}n\phi }+z^{n}\left(I,t\right)e^{-in\phi }\right]\right\},$$ (9)

where *z* (*I*, *t*) is the local order parameter, connected with the global complex order parameter (Eq. 5) *via*

$$Z\left(t\right)=\int_{-\infty }^{\infty }g\left(I\right)z\left(I,t\right)dI.$$ (10)

Inserting Eq. 9 into Eq. 8, one obtains the Ott-Antonsen equation for the layer dynamics

$$\dot{z}=\frac{1}{2}\left(1-z^{2}\right)+iIz+\frac{\sigma }{2}Z-\frac{\sigma }{2}\bar{Z}z^{2},$$ (11)

where bar denotes the complex conjugate.

### 4.2 Stationary Solutions of the Layer Dynamics

To find stationary solutions of Eqs 10, 11, we first write the local order parameter in polar form *z* (*I*, *t*) = *ρ*(*I*, *t*)*e* ^*iϑ*(*I*,*t*)^. Separating for the real and imaginary parts, Eq. 11 becomes

$$\begin{array}{cc} \dot{\rho } & =\frac{1}{2}\left(1-\rho^{2}\right)B\cos\Phi ,\\ \rho \dot{\Phi } & =I\rho -\frac{1}{2}\left(1+\rho^{2}\right)B\sin\Phi , \end{array}$$ (12)

where the new variables *B*, *β* and Φ are given by

$$\begin{array}{cc} B\left(t\right)e^{i\beta \left(t\right)} & =1+\sigma R\left(t\right)e^{i\Theta \left(t\right)},\\ \Phi & =\vartheta -\beta . \end{array}$$ (13)

From Eq. 13, it follows that *B* and *β* are related with the macroscopic order parameter Eq. 5 *via*

$$\begin{array}{cc} B & =\sqrt{1+\sigma^{2}R^{2}+2\sigma R\cos\Theta },\\ \tan\beta & =\frac{\sigma R\sin\Theta }{1+\sigma R\cos\Theta }. \end{array}$$ (14)

Note that the local dynamics can be rewritten in terms of *B* as ${\dot{\phi }}_{k}=I_{k}-B\sin\left(\phi_{k}-\beta \right)$ , suggesting that *B* may be understood as an effective excitability parameter that describes how local excitability is changed by the impact of interactions. As a consequence, the structure of stationary solutions of the Ott-Antonsen system Eq. 12 depends on the relation between |*I*_*k*_| and *B*, such that a population splits into two groups comprised of excitable (|*I*| < *B*) or oscillating units (|*I*| > *B*). In particular, the stationary solutions (*ρ* *^∗^*, Φ*^∗^*) are given by

$$\begin{array}{cc} \left(\rho^{*},\Phi^{*}\right) & =\left(1,\arcsin\frac{I}{B}\right),\\ \left(\rho^{*},\Phi^{*}\right) & =\left(1,\pi -\arcsin\frac{I}{B}\right), \end{array}$$ (15)

for the excitable (inactive) group, and

$$\left(\rho^{*},\Phi^{*}\right)=\left(\frac{|I|-\sqrt{I^{2}-B^{2}}}{B},\frac{\pi }{2}\mathrm{sign}\left(I\right)\right)$$ (16)

for the oscillating (active) group. An explicit expression for *B* can be obtained by invoking the self-consistency relation between the global and local order parameter (Eq. 10). Inserting the results for the stationary local and global order parameter [using Eq. 10, Eq. 15, Eq. 16, and the first equation from Eq. 13] and separating for the real and imaginary parts, one ultimately arrives at a self-consistency equation for *B* (Lafuerza et al., 2010; Klinshov and Franović, 2019)

$$p\left(B\right)=B^{2}-2\sigma p_{2}\left(B\right)+\frac{\sigma^{2}}{B^{2}}\left(p_{1}^{2}\left(B\right)+p_{2}^{2}\left(B\right)\right)-1=0,$$ (17)

where *p*_1_(*B*) and *p*_2_(*B*) are given by

$$\begin{array}{ll} p_{1}\left(B\right) & =r_{1}-\underset{|I|>B}{\int }Ig\left(I-r_{1}\right)\sqrt{1-{\left(\frac{B}{I}\right)}^{2}}\;dI,\\ p_{2}\left(B\right) & =\underset{|I|<B}{\int }g\left(I-r_{1}\right)\sqrt{B^{2}-I^{2}}\;dI. \end{array}$$ (18)

Having determined *B*, the stationary local and global order parameters can be obtained using the relations $R=\sqrt{p_{1}^{2}+p_{2}^{2}}/B$ and Θ = arctan (*p*_1_/(*p*_2_ − *σR* ^2^)), which follow from Eqs 10–14.

**FIGURE 3:**
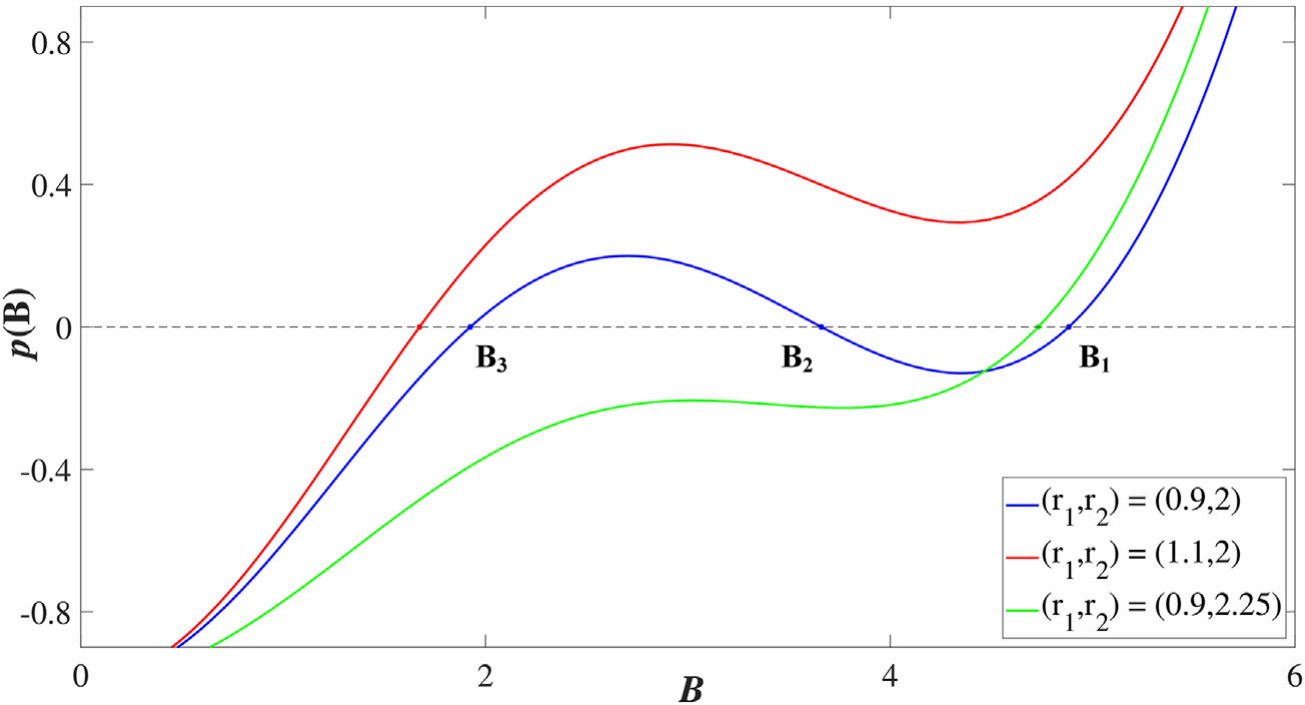
Changes in form and the number of roots of the function *p* (*B*) given by Eq. 17 under variation of *r*_1_ and *r*_2_ for fixed *σ* = 5. The function *p* (*B*) has three roots for (*r*_1_, *r*_2_) = (0.9, 2) (blue line; roots indicated by letters) and a single root for (*r*_1_, *r*_2_) = (1.1, 2) (red) and (*r*_1_, *r*_2_) = (0.9, 2.25) (green).

**FIGURE 4:**
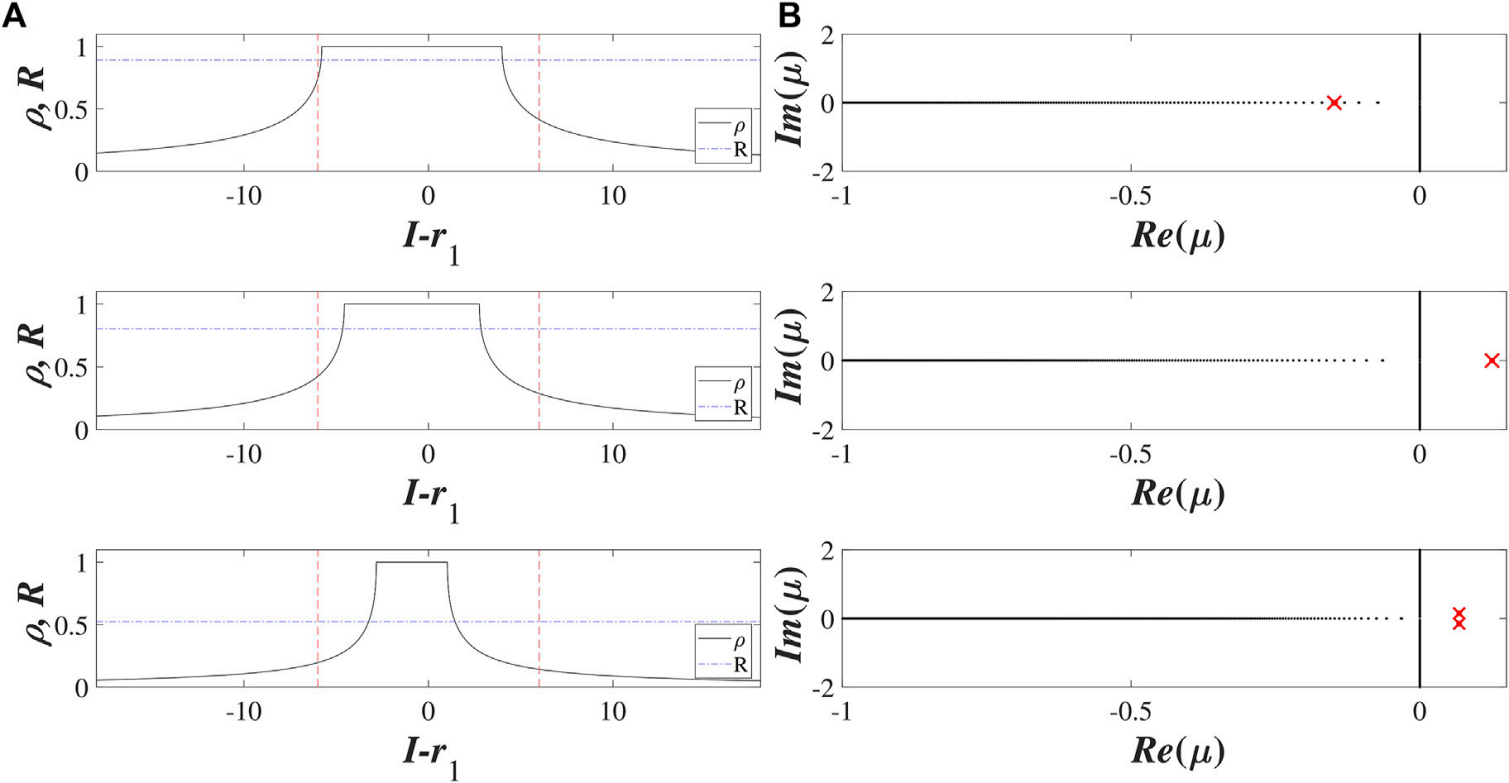
Local structure and spectra of stationary solutions *B*_1_ (a), *B*_2_ (b) and *B*_3_ (c) of Ott-Antonsen equation Eq. 11 for *σ* = 5 and (*r*_1_, *r*_2_) = (0.9, 2). **(A)** shows the dependencies of the local order parameter on the input *z* (*I*) (black solid lines) and the corresponding Kuramoto order parameter *R* (blue dash-dotted lines) for the three stationary solutions. Red dashed lines indicate the interval (*r*_1_ − 3*r*_2_, *r*_1_ + 3*r*_2_) relevant for the distribution of external inputs. **(B)** shows the continuous (black dots) and the discrete spectra (red crosses) for the stationary solutions: *B*_1_ and *B*_2_ are stable and unstable nodes, respectively, while *B*_3_ is an unstable focus.

For a fixed coupling strength *σ*, the function *p*(*B*) may have from one to three roots, depending on the mean value *r*_1_ and the standard deviation *r*_2_ of the distribution of intrinsic parameters *I*_*k*_. The examples in Figure 3 illustrate how the number of solutions of Eq. 17 changes between one and three for fixed *σ* = 5, *r*_1_ = 0.97 under increasing *r*_2_. We refer to the stationary solutions by the corresponding *B* values, which we arrange in decreasing order *B*_1_ > *B*_2_ > *B*_3_. Recalling the arguments above, one sees that the larger *B* value implies a prevalence of excitable over oscillating units within the local structure of the stationary state. This is evinced by the left column of Figure 4 which shows the dependence of the local order parameter *z*(*I*). Typically, the state *B*_1_ comprises of a clear majority of excitable units, corresponding to a coherent domain *z* = 1, and may thus be referred to as a *homogeneous* stationary state. The two remaining stationary states *B*_2_ and *B*_3_ are *heterogeneous* in the sense that they involve a mixture of excitable and asynchronously oscillating units.

### 4.3 Stability and Bifurcation Analysis of Stationary Solutions

Given that Ott-Antonsen equation Eq. 11 contains both the global order parameter and its complex conjugate, stability and bifurcation analysis of the stationary solutions (Omel’chenko and Wolfrum, 2013; Klinshov and Franović, 2019) can be carried out by writing the local and global order parameters as *z* (*I*, *t*) = *x* (*I*, *t*) + i*y* (*I*, *t*), *Z*(*t*) = *X*(*t*) + i*Y*(*t*) and separating for the real and imaginary parts. This results in the system

$$\begin{array}{cc} \dot{x} & =F\left(x,y,X,Y\right)=\frac{1}{2}\left(1-x^{2}+y^{2}\right)-Iy+\frac{\sigma }{2}X-\frac{\sigma }{2}X\left(x^{2}-y^{2}\right)-\sigma xyY,\\ \dot{y} & =G\left(x,y,X,Y\right)=-xy+Ix+\frac{\sigma }{2}Y-\sigma xyX+\frac{\sigma }{2}Y\left(x^{2}-y^{2}\right), \end{array}$$ (19)

which can be linearized for variations *ξ* = (*δx*, *δy*)^*T*^, Ξ = (*δX*, *δY*)^*T*^ around the stationary solution (*x*_0_, *y*_0_, *X*_0_, *Y*_0_), ultimately arriving at

$$\frac{d\xi }{dt}=\hat{P}\left(I\right)\xi \left(I,t\right)+\hat{Q}\left(I\right)\Xi \left(t\right),$$ (20)

where $\hat{P}$ and $\hat{Q}$ are the corresponding Jacobian matrices

$$\hat{P}=\left(\begin{array}{cc} \frac{\partial F}{\partial x} & \frac{\partial F}{\partial y}\\ \frac{\partial G}{\partial x} & \frac{\partial G}{\partial y} \end{array}\right),\hat{Q}=\left(\begin{array}{cc} \frac{\partial F}{\partial X} & \frac{\partial F}{\partial Y}\\ \frac{\partial G}{\partial X} & \frac{\partial G}{\partial Y} \end{array}\right).$$

Eq. 20 is augmented by the variational equation for Eq. 10:

$$\Xi \left(t\right)=\int_{-\infty }^{\infty }g\left(I\right)\xi \left(I,t\right)\;dI.$$ (21)

Assuming that the variations *ξ* (*I*, *t*) and Ξ (*t*) satisfy *ξ* (*I*, *t*) = *ξ*_0_ (*I*)*e* ^*μt*^, Ξ (*t*) = Ξ_0_ *e* ^*μt*^, systems Eq. 20 and Eq. 21 transform into

$$\left(\hat{P}\left(I\right)-\mu \hat{I}\right)\xi_{0}\left(I\right)+Q\left(I\right)\Xi_{0}=0,\;\Xi_{0}=\int_{-\infty }^{\infty }g\left(I\right)\xi_{0}\;dI,$$ (22)

where $\hat{I}$ denotes the identity operator. From the general spectrum theory of linear operators (Omel’chenko and Wolfrum, 2013; Mirollo and Strogatz, 2007), it follows that the Lyapunov spectrum of Eq. 22 consists of a continuous and a discrete part. Here, the continuous spectrum turns out to be always stable or marginally stable, such that the stability of stationary solutions depends on the discrete spectrum. The latter can be determined by rewriting Eq. 22 in the form $\hat{C}\left(\mu \right)\Xi_{0}=0$ , where

$$\hat{C}\left(\mu \right)=\hat{I}+\int_{-\infty }^{\infty }g\left(I\right){\left(\hat{P}\left(I\right)-\mu \hat{I}\right)}^{-1}Q\left(I\right)\;dI.$$ (23)

The discrete spectrum is then obtained by solving the characteristic equation $\det\hat{C}\left(\mu \right)=0$ (Klinshov and Franović, 2019). An example of the discrete and continuous spectra calculated for the stationary states *B*_1_, *B*_2_ and *B*_3_ at (*r*_1_, *r*_2_) = (0.9, 2) is provided in the right column of Figure 4.

### 4.4 Comparison Between Analysis and Numerics

**FIGURE 5:**
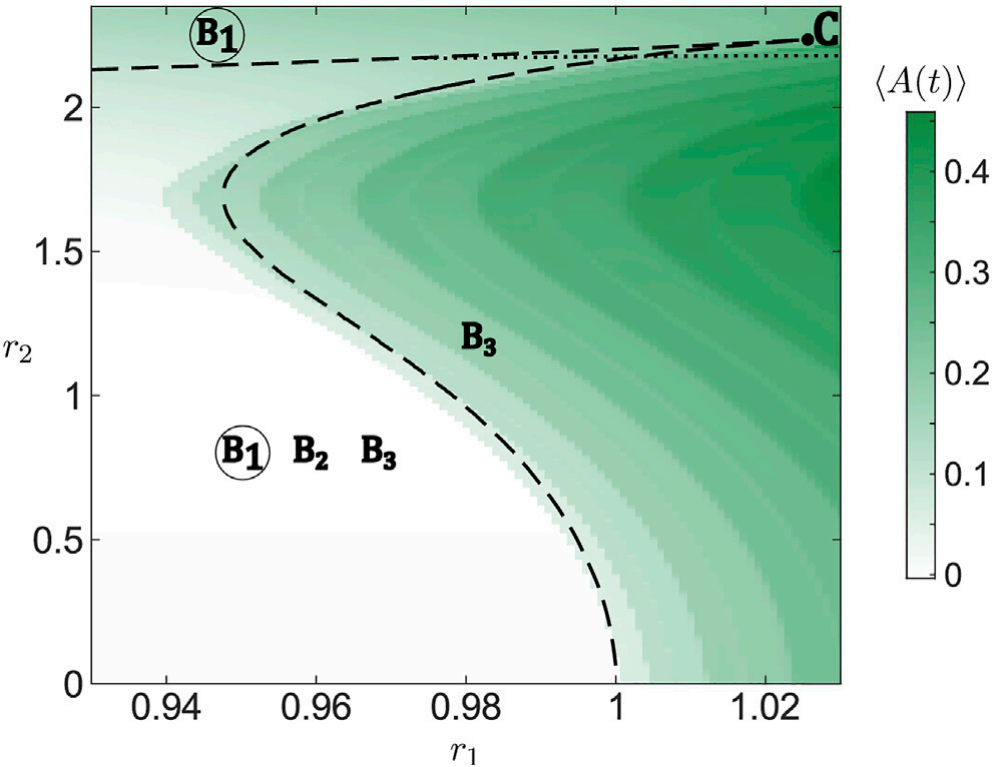
Bifurcation diagram for the system of active rotators Eq. 1 in dependence on resource levels *r*_1_ and *r*_2_. For the simulation, we have chosen one set of random initial conditions for the phases and one set of parameters *ν*_*k*_ randomly drawn from a normalized Gaussian distribution N(0,1) . Simulations comprise of 200 time units with activity averaged over the last 100 time units. Fold bifurcations involving stationary solutions *B*_1_ and *B*_2_ (lower branch) and *B*_2_ and *B*_3_ (upper branch) obtained from Eq. 17 are shown by black dashed lines that give rise to a cusp point marked with *C*. Existence of particular solutions and their stability, derived from the discrete spectrum of Eq. 23, are indicated by their corresponding letters and a circle, respectively, whereby the circle indicates a stable solution. Along the black dotted line, stationary solution *B*_3_ changes its stability in a Hopf-like bifurcation. Other parameters: *N* = 5,000, *σ* = 5.

The previous analysis allows for an analytic description of the existence and stability of stationary solutions in the limit of large populations (*N* → *∞*). In particular, the bifurcation diagram for the Ott-Antonsen equation of the layer dynamics Eq. 11 in the (*r*_1_, *r*_2_) plane is organized around a co-dimension two cusp point, indicated by C in Figure 5 where the two branches of folds meet (black dashed lines). Both branches of folds are calculated by numerical continuation of the solutions of Eq. 17 using the software package BifurcationKit.jl (Veltz, 2020). The lower branch of folds which folds over for larger *r*_2_ corresponds to annihilation and reemergence of a pair of equilibria, *B*_1_ and *B*_2_, whereby the former (latter) is always stable (unstable). For smaller *r*_2_, crossing this branch either by enhancing *r*_1_ or *r*_2_ gives rise to long-period collective oscillations as a stable equilibrium *B*_1_ vanishes by colliding with an unstable equilibrium *B*_2_. The divergence of the oscillation period when approaching the curve indicates that it corresponds to a SNIPER bifurcation of the full system. For larger *r*_2_, as the branch folds over, one observes the reappearance of a stable stationary state *B*_1_, emerging in an inverse fold bifurcation together with an unstable equilibrium *B*_2_. The upper branch of folds involves stationary states *B*_2_ and *B*_3_, such that they collide and disappear above the curve, where *B*_1_ remains the only stable stationary state, cf. Figure 5.

Note that apart from the fold bifurcation, the stability of *B*_3_ is also affected by a Hopf-like bifurcation (black dotted line). Above the given curve, stability of *B*_3_ is determined by a pair of complex conjugate eigenvalues which have the smallest negative real parts. However, crossing the curve, these eigenvalues merge with the imaginary axis and remain neutrally stable immediately below the curve, implying that the central manifold theorem associated to Hopf bifurcation cannot immediately be applied. Still, in close vicinity below the curve, starting from an initial condition corresponding to *B*_3_ results in oscillations similar to a genuine scenario of Hopf bifurcation.

Using numerical continuation, we have verified that the described structure of bifurcation diagram for the layer dynamics remains qualitatively the same under variation of coupling strength *σ*. One only notes that for increasing *σ*, the branches of folds shift toward larger *r*_2_, which corresponds to a higher diversity of external inputs.

Figure 5 further shows a comparison of the existence and stability conditions for the collective stationary states derived from Ott-Antonsen approach for the limit *N* → *∞* with simulations for a finite population of *N* = 5,000 active rotators with fixed resources *r*_1_ and *r*_2_. One observes that simulation results agree well with the fold bifurcation lines separating parameter regimes of low and high collective activity. The differences can be attributed to the finite size of assemblies considered in the simulations.

**FIGURE 6:**
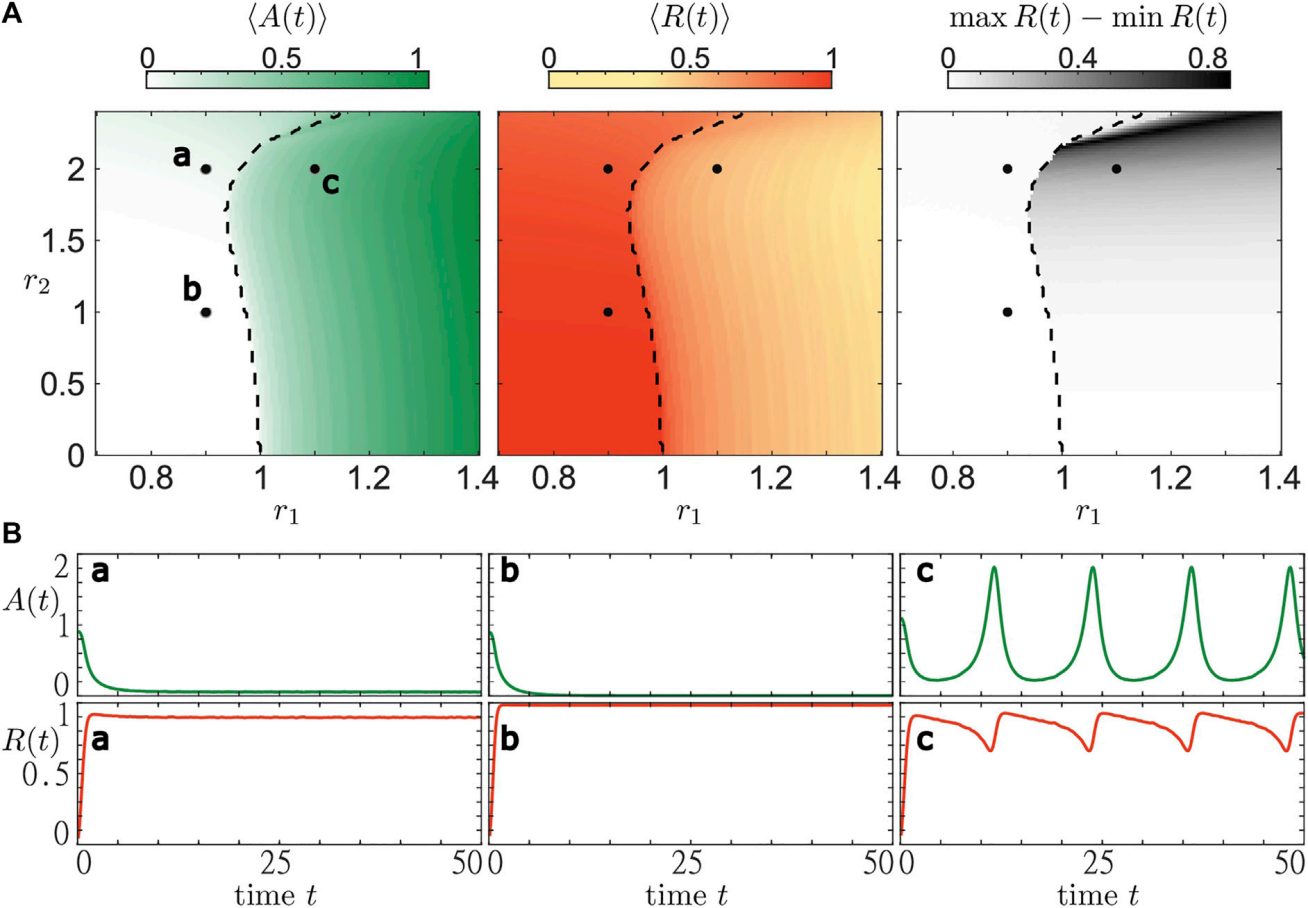
Bifurcation diagram for the system of active rotators Eq. 1 in terms of resource levels (*r*_1_, *r*_2_). All simulations are carried out the same way as in Figure 5. Three diagrams at the **(A)** show the time-averaged values of activity *A* (*t*) (white-green), order parameter *R*(*t*) (yellow-red) and variations of the order parameter max *R*(*t*) − min *R*(*t*) (white-black). The black dashed line separates regions where the mean phase Θ of the complex order parameter *Z* features stationary or oscillating dynamics, respectively. The **(B)** show time traces of activity and order parameter for three parameter pairs (*r*_1_, *r*_2_): a—(0.9,2), b—(0.9,1), c—(1.1,2).

With Figure 6 we complement the analysis of the layer equation. In particular, we show how the dynamical regimes change in a wide range of parameters *r*_1_ and *r*_2_, and indicate the boundary (black dashed line) that separate parameter regions supporting stable stationary states from those admitting oscillatory states. We illustrate three different trajectories corresponding to qualitatively different collective regimes found by numerical analysis. For parameter pairs *a* and *b*, we observe the emergence of stationary states in accordance with the bifurcation analysis shown in Figure 5. In both cases, activity *A* (*t*) and the coherence measure *R* (*t*) settle to a constant value. We observe that with increasing *r*_2_ (*b* to *a*) the activity level rises while the coherence level declines. For the parameter set *c*, there is no stable stationary state and we observe stable oscillations. The activity shows a regular, tonic-like spiking shape corresponding to an increase in the average activity. Meanwhile, variation of the order parameter causes its average value to decrease. In order to quantify the temporal variations of the order parameter, we also plot the difference max  *R* (*t*)−min *R* (*t*) for the considered average time interval. We observe that even though the activity level might be high, e.g. for *r*_1_ > 1 close to *r*_2_ = 0, the coherence within the population is not necessarily strongly varying. However, there are also regimes, e.g. for *r*_1_ > 1 and *r*_2_ > 2, where the order parameter varies strongly and covers almost the entire interval from 0 to 1. In this section, we have illustrated the stability regions of macroscopic stationary states in a heterogeneous population of active rotators. Numerically, we have also determined the values of resource parameters *r*_1_ and *r*_2_ where no stable stationary solutions exist. Using these insights, we are now able to qualitatively describe the phenomena in systems with a slow adaptation of the resources and the resource-dependent dynamics. The next section is devoted to explaining the emerging states of collective activity bursting.

## 5 (Slow) Resource Dynamics and the Emergence of Multistability

The analysis of the layer dynamics in Section 4 provides insight on how the system evolves for constant resources *r*_1_ and *r*_2_. Due to the different timescales of the population (fast dynamics) and the pool of resources (slow dynamics), we can average (Sanders et al., 2007; Franović et al., 2020) the system Eqs 2, 3 as

$$\dot{r}=f\left(r-s,\lambda \right),$$ (24)

$$\dot{\lambda }=-\lambda +\lambda_{0}+\rho \left\langle A\right\rangle ,$$ (25)

where $\left\langle A\right\rangle =\frac{1}{T}\int_{0}^{T}A\left(s\right)ds$ . Here we also assume *ϵ*′ = *ϵ* and rescale time *t*_new_ = *ϵt*_old_. Note that the average activity shown in Figure 6 depends on the resource variables ***r***, since the definition Eq. 4 implies *A* = *r*_1_ − Im (*Z*). Hence, the system Eqs 24, 25 describes an effective three-dimensional coupled dynamics for the slow subsystem. A further analytical analysis of this system is beyond the scope of our study. However, we directly use the insight that the slow dynamics follows the average activity of the fast system to understand the emergence of collective activity bursting.

**FIGURE 7:**
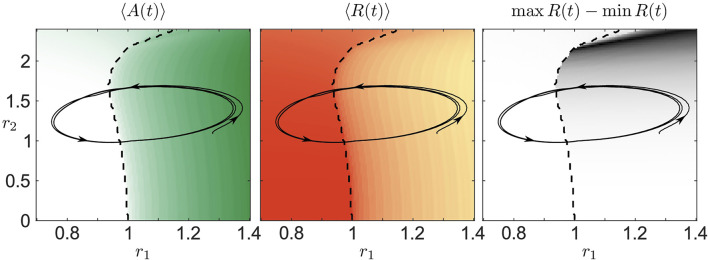
Bifurcation diagrams as in Figure 6 complemented with the trajectories of the resource variables *r*_1_ (*t*) and *r*_2_ (*t*) for the collective activity bursting shown in Figure 6.

Figure 7 shows the trajectories of the resource variables *r*_1_(*t*) and *r*_2_ (*t*) for the collective bursting presented in Figure 6 along with the averaged values of population activity and the order parameter. We clearly see that the asymptotic orbit passes through both the regimes of an active and inactive population which explains the episodes of high and low activity in Figure 2A. Also the segments of increasing and decreasing average activity visible in Figure 2A can be explained by Figure 7. Here, the average activity shows the same pattern along the trajectory (*r*_1_ (*t*), *r*_2_ (*t*)). The same also holds for the average values of the order parameter and even its variations if we compare Figure 2B with Figure 7. Therefore, the splitting of the fast from the slow subsystem provides a very good qualitative explanation for the observed phenomenon. To understand the emergence of the full periodic orbit shown in Figure 7, we first note that without any population activity, i.e., ⟨*A*⟩ = 0 and *λ*_0_ = − 0.05, the resource dynamics possess a stable focus close to the critical line (black dashed line in Figure 7) describing the transition from stationary to oscillatory dynamics of the mean phase Θ. During the stationary phase, *r*_1_ and *r*_2_ tend to *s*_1_ = 0.97 and *s*_2_ = 1.2, respectively. As in Figure 7, the trajectory (*r*_1_ (*t*), *r*_2_ (*t*)) may start in the active region, i.e., oscillatory mean phase dynamics. Due to the positive average value of activity, the variable *λ* (*t*) characterizing the resource activity increases according to (Eq. 25) and becomes positive, see Figure 2C. Hence, the resources become activated and (*r*_1_(*t*), *r*_2_(*t*)) follows the limit cycle solution of the resource dynamics revolving around (*s*_1_, *s*_2_). Note that the resources obey the Hopf normal form with a Hopf bifurcation at *λ* = 0, see Eq. 2. After passing the critical line, the average activity immediately drops to ⟨*A*⟩ ≈ 0 which causes *λ* to tend to *λ*_0_, see Figure 2C. After *λ* falls below zero, the dynamics of the resources (*r*_1_(*t*), *r*_2_(*t*)) is described by a spiral towards (*s*_1_, *s*_2_). This spiral, however, enters the active region by passing the critical line which ultimately leads to the recurrent phenomenon observed in Figure 2. As we have seen, the emergence of collective activity bursting relies on the subtle interplay between activation and deactivation of resources and the population. Furthermore, the need for the spiraling dynamics towards a stable focus explains well the necessity for the resource basis levels (*s*_1_, *s*_2_) to be close to the critical line separating the population active and inactive regimes.

With regards to the above description of the collective activity bursting, one might ask for the coexistence of a stable steady state in the system Eqs 1–3 as long as (*s*_1_, *s*_2_) lie in the inactive regime. This state might have a small basin of attraction such that the spiral towards the steady state cannot reach the active regime.

**FIGURE 8:**
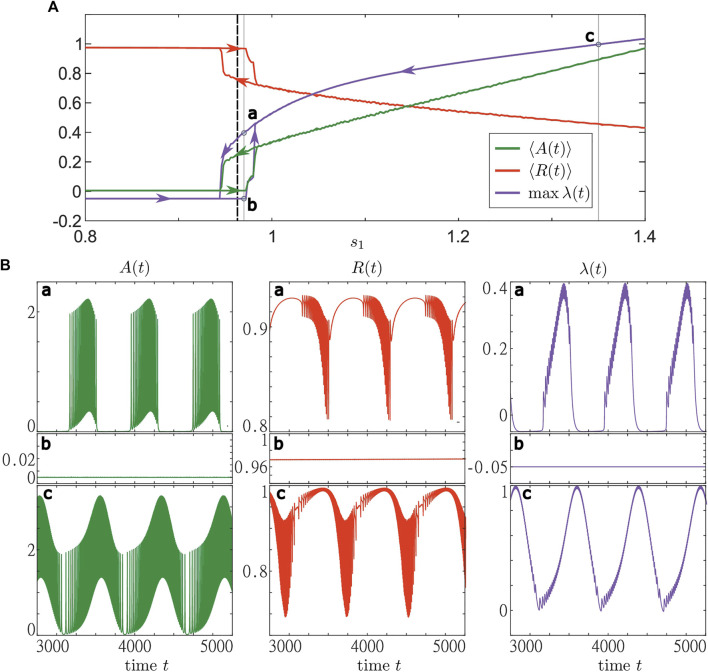
Bifurcation diagram with respect to the resource base level *s*_1_ for a system of active rotators with adaptive resource interaction Eqs 1–3. The **(A)** shows the results from two adiabatic continuations with step size Δ*s*_1_ = 0.002 from *s*_1_ = 0.8 to *s*_1_ = 1.4 (sweep up) and vice versa (sweep down). Sweeps up and down start from a stable stationary and a stable oscillatory state, respectively. For both sweeps are shown the average activity ⟨*A*(*t*)⟩ (green), average order parameter ⟨*R*(*t*)⟩ (red) and maximal resource activity *λ* (blue). The results were obtained by simulating (1)–(3) for 7,000 time units and taking the average over the last 5,000 time units. The branches corresponding to the two sweeps are marked by arrows. The black dashed lines indicate the value (*s*_1_ ≈ 0.963) of the critical line shown in Figure 6. Three trajectories represented by the activity (green, first column), order parameter (red, middle column) and the resource activity (blue, last column) are shown in the **(B)** for (a,b) *s*_1_ = 0.97 and (c) *s*_1_ = 1.35. The panels in (a) and (b) represent the states found along the sweeps down and up, respectively. The simulations were performed using the same values of *ν*_*k*_ as in Figure 5. Parameters: *N* = 5,000, *σ* = 5, *ϵ* = 0.05, *s*_2_ = 1.2, *ω* = 0.2, *γ* = 0.5.

In order to get insights into the different stable states that exist in Eqs 1–3, we use the numerical method of adiabatic continuation. To do so, we fix the base level *s*_2_ = 1.2 and gradually vary *s*_1_ from 0.8 to 1.4 (sweep up) and from 1.4 to 0.8 (sweep down). For each value of *s*_1_, we run the simulation starting from the final state of the previous simulation. In Figure 8, we show the results of both sweeps. We observe the existence of stable steady and stable oscillating states for various values of *s*_1_. As expected, close to the boundary between active and inactive states of layer dynamics, we also find an interval of coexistence between collective activity bursting and stable steady states, see panels for (a) and (b) in Figure 8, respectively. For larger *s*_2_, only the oscillatory state can be observed, which does not enter the inactive regime above a certain *s*_1_, see panel (c) in Figure 8. Note that the character of the solution can be deduced from the maximal value of *λ*(*t*) on the averaging time interval. In particular, there is a stationary state only if max *λ*(*t*) < 0. In all other cases, there are time intervals where the trajectory of ***r*** diverges from the base level ***s*** and follows the periodic solution of Eq. 24.

From the arguments laid out in this section, we have seen that the mutual activation and deactivation between the neural population and the pool of resources close to criticality of layer dynamics induces a rich dynamical behavior. It is believed, particularly, that the human brain operates close to criticality (Chialvo, 2010; Haimovici et al., 2013; Yu et al., 2013; Cocchi et al., 2017; Wilting and Priesemann, 2019). Therefore, it is of major importance to understand the dynamics of neural populations in this regimes including the interaction with its environment. In the next section, we propose a simple mechanism which can induce a switch between coexisting macroscopic regimes.

## 6 Population Switching Dynamics Induced by Resource Activation and Inactivation

In the vicinity of the transition between the population inactivity and activity, we have observed collective activity bursting induced by an adaptive dynamical pool of resources. Moreover, this phenomenon emerges in a stable coexistence with a steady state. In this section, we consider two simple perturbation approaches that can induce a switch between these two functionally different states.

**FIGURE 9:**
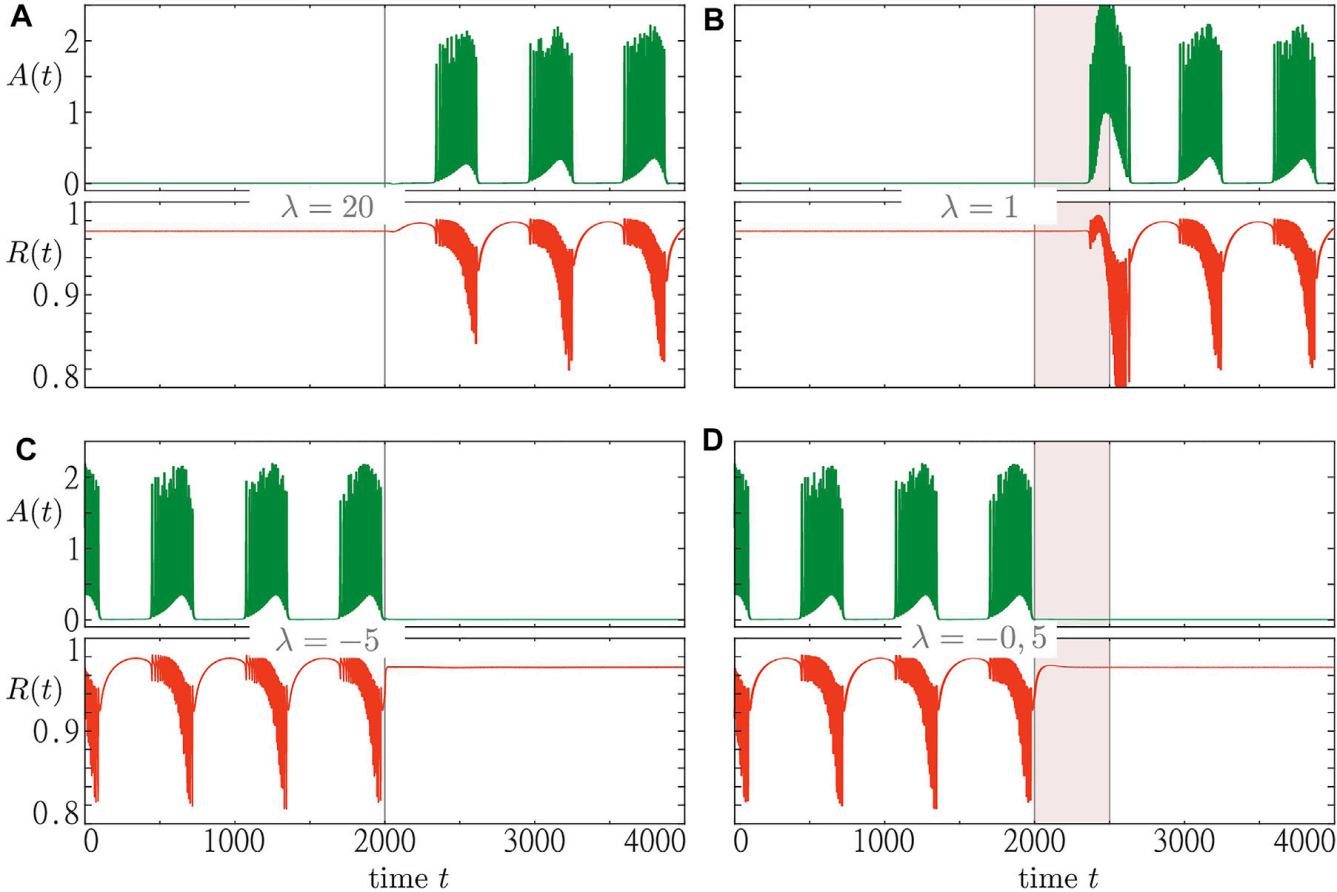
Two perturbation scenarios to induce a switch between an inactive steady state and collective activity bursting. Time traces of macroscopic activity *A* (*t*) and order parameter *R* (*t*) are shown green and red, respectively. In the panels **(A)** and **(B) (C,D)**, we start from an initial steady state (bursting state). The first perturbation scenario is illustrated for the cases where the resource activity variable *λ* is set to *λ* = 20 [panel **(A)**] and *λ* = −5 [panel **(C)**] at *t* = 2000. The second perturbation scenario is demonstrated for the cases where the resource activity is kept fixed at *λ* = 1 [panel **(B)**] and *λ* = −0.5 [panel **(D)**] for a duration of 500 t. u. beginning at *t* = 2000 t. u. Simulations were run for *s*_1_ = 0.97 and the remaining parameters fixed as in Figure 8.

Figure 9 shows the results for two different perturbation approaches to system Eqs. 1–3. The first approach aims to induce a switch in population dynamics by an instantaneous resetting of the resource activity *λ*. In the second approach, we induce such a transition by maintaining the resource activity at a certain level for a certain period of time. The first approach works well for large resetting values of *λ*, see Figures 9A,C. Small values, however, would not be sufficient to induce the macroscopic regime switch. Furthermore, in case of an initial bursting state, eliciting the switch to a steady state depends on the moment at which the perturbation is applied. However, in our numerical simulations (not shown), we have always been able to induce a switching for sufficiently large resetting values of *λ*.

Due to the functionally very different nature of the two stable states, there might be reasons to favor one over the other in light of potential applications in medicine. Therefore, it is of great interest to understand simple mechanisms that would induce a switch to the desired state. While the first perturbation approach provides such a mechanism, it still requires strong perturbations which might be undesirable for certain medical reasons, e.g. side effects. Therefore, we have proposed another perturbation approach that leads to a switch while keeping the reset level lower. For this approach, we have also been able to induce switches between a steady state and a bursting state in one or the other direction, see Figures 9B,D, with the advantage of having the resetting level of the resource activity much lower than for the first method.

In this section, we have proposed two simple perturbation approaches to induce a switch between the two functionally different macroscopic states of the full system which emerge near the transition in layer dynamics between the population activity and inactivity and due to an adaptive dynamical pool of resources. We note that the approaches we proposed are not the only way to induce macroscopic regime shifts. One might also think of perturbing the resource variables (*r*_1_, *r*_2_) or even the whole population. Thus, perturbation of the resource activity variable is perhaps the simplest but not the only approach possible.

## 7 Conclusion

We have investigated collective dynamics in a system of interacting excitable units coupled to a pool of resources with nontrivial dynamics. The feedback of the resources to the population of coupled excitable units has been realized by an adaptation of the individual units’ inputs, whereas in turn, the excitable population is capable of activating or deactivating the pool of resources depending on the population’s own activity. As a prototype of excitable local dynamics, we have considered active rotators. Following the ideas outlined by Roberts et al. (Roberts et al., 2014), we have assumed the processes at the pool of resources to occur much slower than the local dynamics of excitable units. As a consequence, we have ended up with a system featuring multiscale dynamics, allowing us to use the methods from singular perturbation theory (Desroches et al., 2012; Kuehn, 2015).

As our most important finding, we have reported on the phenomenon of collective activity bursting. The phenomenon is characterized by a recurrent switching between episodes of quiescence and episodes of activity bursts in the population of active rotators. To gain a better understanding of the emergence of collective activity bursting, we have made use of the explicit slow-fast timescale separation. In particular, we have divided the system dynamics into the fast layer dynamics of the population and the slow average dynamics of the resources.

Using the Ott-Antonsen approach, we have analyzed the stability and bifurcations of the stationary solutions of layer dynamics in the thermodynamic limit. For the population of active rotators with a heterogeneity given by a Gaussian distribution, we have derived a bifurcation diagram for the steady state solutions. The bifurcations of layer dynamics depending on the mean and the width of the Gaussian distribution have been corroborated by numerical simulations of a large ensemble of rotators. Doing so, we have determined the parameter regions admitting high or low (or even no) population activity and have obtained the critical lines separating these regions.

Taking the analysis of the layer problem into account, we have further analyzed how the slow averaged dynamics of the resources gives rise to a slow variation of the mean and width of the Gaussian distribution. We have observed the onset of collective activity bursting close to criticality where the population of active rotators undergoes a transition from an inactive to an active state. The emergence of collective bursting is due to a subtle interplay of co-activation and co-deactivation of the dynamical population of rotators and the pool of resources.

We have further found a region of bistability between collective activity bursting and an inactive steady state close to criticality of the layer dynamics. A similar observation has been also discussed in the context of collective bursting induced by synaptic short-term plasticity (Gast et al., 2020). Moreover, we have proposed two different perturbation methods that can trigger switches between coexisting macroscopic regimes. In particular, we have demonstrated that the regime shifts can be induced either by using instantaneous large perturbations or persistent perturbations of the resource activity.

In terms of theory, an important extension of our work could concern a further analytical study of the reduced slow-fast system governing the collective dynamics of the ensemble of excitable units and its interaction with the resources. For convenience, we summarize the reduced system here

$$\begin{array}{ll} \dot{z}\left(I,t\right) & =\frac{1}{2}\left(1-z^{2}\left(I,t\right)\right)+iIz\left(I,t\right)+\frac{\sigma }{2}Z\left(t\right)-\frac{\sigma }{2}\bar{Z}\left(t\right)z^{2}\left(I,t\right),\\ \dot{r}\left(t\right) & =\epsilon f\left(r\left(t\right)-s,\lambda \left(t\right)\right),\\ \dot{\lambda }\left(t\right) & =-\epsilon \left[\lambda \left(t\right)-\lambda_{0}-\rho \left(r_{1}-\text{Im}\left(Z\left(t\right)\right)\right)\right], \end{array}$$

with

$$Z\left(t\right)=\int g\left(I\right)z\left(I,t\right)\;dI.$$

In a broader context, we have proposed a simple paradigmatic model to study the emergence of complex collective phenomena induced by a dynamically co-evolving pool of resources. The research on the impact of resource constraints on the dynamical regimes of populations of neurons or neuron-like units from the dynamical network perspective (Nicosia et al., 2017; Kroma-Wiley et al., 2021) has begun only recently. In our study, we have shown that even a simple model that includes nontrivial dynamical resources gives rise to the emergence of collective activity bursting close to criticality in a population of neuron-like excitable units. Our study underlines the potentially important role of resource constraints in the operating of the human brain that is often hypothesized to operate close to criticality. We have further shown that the collective activity bursting may stably coexist with a steady state. Either one of these regimes could be desirable or undesirable, which makes understanding of the control mechanisms to switch between the regimes highly important (Tang and Bassett, 2018). In this context, we have discussed two simple approaches that can successfully induce such regime shifts. Both approaches impose perturbations to the single activity variable of the resource pool and can thus be generalized to systems with even more complex dynamical resource pools.

## Data Availability

The original contributions presented in the study are included in the article/Supplementary Material, further inquiries can be directed to the corresponding authors.
